# Feasibility and accuracy of single time point imaging for renal dosimetry following ^177^Lu-DOTATATE (‘Lutate’) therapy

**DOI:** 10.1186/s40658-018-0232-9

**Published:** 2018-12-20

**Authors:** Kathy P. Willowson, Enid Eslick, Hyunju Ryu, Aurora Poon, Elizabeth J. Bernard, Dale L. Bailey

**Affiliations:** 1 Department of Nuclear Medicine, Royal North Shore Hospital, St Leonards, NSW 2065 Australia; 2 Institute of Medical Physics, The University of Sydney, Camperdown, NSW 2006 Australia; 3 Faculty of Health Sciences, The University of Sydney, Lidcombe, NSW 2141 Australia; 4 Department of Molecular Imaging and Therapy, The Austin Hospital, Heidelberg, VIC 3084 Australia

**Keywords:** Lutate, Dosimetry, Kidney, Quantitative, SPECT/CT

## Abstract

**Background:**

This study aims to assess both feasibility and accuracy of renal dosimetry imaging protocols in patients receiving Lutate therapy for neuroendocrine tumours (NETs), when data acquisition over multiple days is not possible on all cycles.

**Method:**

Patients who had received a full 4 cycles of Lutate therapy with complete imaging at each cycle were included. Imaging consisted of quantitative SPECT/CT of the kidneys at 4, 24 and 96–120 h post injection. Renal absorbed dose was calculated for each data set, and in addition, five alternative methods were explored for comparison. Method 1: a patient average clearance time (*t*_1/2_ average) derived from the first half of contributing patient data was used to estimate absorbed dose for subsequent patients based on 4 h imaging alone; method 2: *t*_1/2_ average was applied to subsequent patients on 24 h imaging alone; method 3: a patient-specific clearance rate (*t*_1/2_ patient) was determined from complete image data of cycle 1 and applied subsequently to remaining cycles using 4 h image data alone; method 4: *t*_1/2_ patient was applied to 24 h imaging alone in subsequent cycles; method 5: the 120 h data was estimated on subsequent cycles based on the cycle 1 fraction of injected activity (%IA) at 24 and 120 h.

**Results:**

Twenty treatments from 18 patients, resulting in 80 cycles of therapy, were analysed. The measured average renal absorbed dose per cycle of treatment was 0.38 ± 0.19 Gy/GBq when derived from full imaging data. The use of *t*_1/2_ average applied to a single time point led to large deviations of dose estimates from true values (on average 59% and 30%, when using 4 h data and 24 h data, respectively). The use of complete image data on cycle 1 and the derivation of *t*_1/2_ patient led to improved dose estimates, with an average deviation from true values of 13% and 2% when using 4 h data only and 24 h data only, respectively. The use of a 120 h %IA derived from cycle 1 led to an average deviation from true dose estimates of 14%.

**Conclusion:**

In instances where demands on both patients and facilities make multiple time point data acquisition impractical, renal dosimetry is best derived through complete imaging at cycle 1 only followed by a single 24 h imaging time point on subsequent cycles, assuming no significant changes in renal function during the time course of therapy.

## Background

The peptide DOTA-(Tyr^3^)-octreotate labelled with the radionuclide Lutetium-177 (^177^Lu), commonly called ‘Lutate’, is a somatostatin analogue used to selectively target and treat neuroendocrine tumours (NETs) that demonstrate an overexpression of somatostatin receptors. ^177^Lu has a 6.647 day physical half-life and emits β^−^ particles (*E*_max_ = 0.50 MeV) and γs (113 keV [6%] and 208 keV [10%]) [1], which allow for imaging and hence subsequent dosimetry estimates. Lutate is typically delivered over 4 cycles, usually given at 8-week intervals as an injection of 5.5–7.4 GBq [2]. The biokinetics demonstrates rapid blood clearance and urinary excretion, and as such, the organs at risk are considered to be bone marrow and kidney. Whilst renal dosimetry is recommended for all patients receiving treatment, there are several limitations on ability and accuracy to achieve this end point. The non-uniform irradiation of the kidney is problematic from both the perspective of understanding organ toxicity risks as well as limitations of instrumentation spatial resolution and the associated ability to distinguish between uptake in the proximal tubuli (radioresistant) and glomeruli (radiosensitive) [3]. Dose estimates tend to rely on models which may or may not account for patient-specific organ mass which can lead to the incorrect derivation of actual absorbed dose. Furthermore, the ability to accurately derive quantitative images and acquire knowledge on injected radioactivity within the kidneys themselves is also fraught with difficulty and performed with much variation between centres [4]. Aside from difficulties associated with the quantification process itself, one of the more challenging aspects of patient-specific Lutate renal dosimetry relates to the ability of both the clinical department and the patient to fulfil multiple time point image acquisitions, especially on later days, particularly when considering that each complete treatment consists of several cycles of therapy. Whether 2D or 3D imaging is being performed, it is well recognised that three to four time points should ideally be acquired to establish accurate time-activity curves (TACs) fitted with either an exponential, or some variation of, to establish absorbed dose estimates [5]. Typical time points for acquisition are 4 and 24 h, with at least one additional late time point (120 h at our institute), where the late time point imaging is vital to model correct clearance and avoid the assumption of extrapolation from very early time points or physical decay only and so over-estimates of absorbed dose. Aside from the constraints this places on staff and facilities in a busy nuclear medicine clinic, it is also very demanding for patients, particularly those who may not live locally and cannot easily return for scanning at late time points.

Alternatives have been suggested to try and reduce the imaging burden on patients, such as a hybrid 2D/3D imaging approach [6–8]. This method uses multiple planar acquisitions which require less imaging time and only a single SPECT acquisition that is used to scale the TAC for the volume of interest. However, the choice of time point for the SPECT acquisition will affect results, and the assumption of a fixed spatial radioactivity distribution within the target and comparable kinetics from planar and tomographic imaging is questionable [9]. Another method that may benefit patients is that of limited duration SPECT acquisitions aimed at satisfactory quantification as opposed to clinical diagnostic imaging, to obtain multiple time point SPECT data for dosimetry [10]. However, neither of these approaches address the issue of patients returning to the clinic on subsequent days following their treatment. Heikkonen et al. have demonstrated the feasibility of using two SPECT/CT scans (at 24 and 168 h) to estimate kidney dosimetry following Lutate treatment with acceptable accuracy when compared to estimates derived from three time points [11], which they recommend be performed after each treatment cycle. This does still represent a large burden on clinical facilities, and in our experience, it is the late time point that is the most difficult for patients to return for. A recent study by Sundlöv et al. [8] explored possible methods to simplify renal dosimetry through abbreviated scanning and dosimetry protocols, concluding that the use of a single SPECT/CT at 96 h acquired every cycle in conjunction with planar data was sufficient to derive reliable renal dose estimates, highlighting the importance of a late imaging time point. This has been further advocated by Hänscheid et al. [12] who reported on a simplified method requiring only a single quantitative SPECT/CT acquired 4 days after administration of Lutate to derive adequate estimates of organ absorbed dose. Garske et al [13] demonstrated the feasibility of using complete imaging at cycle 1 to derive an effective half-life for an individual patient that could then be used to estimate TACs at subsequent cycles based on only 24 h imaging [13]. Whilst changes in tumour burden and patient disease status may alter effective half-life at later cycles, this technique offers a compromise between patient-specific measures and simplified dosimetry for patients that are not at high risk of renal toxicity.

The aim of this retrospective study was to compare various methods of deriving renal dose estimates in Lutate patients when full imaging data sets are not available and so propose an alternative imaging protocol for clinics that are unable to perform multiple time point image acquisitions for all treatment cycles.

## Methods

Our centre performs 4 cycles for each course of Lutate treatment, each of a standard ~ 8 GBq. The Lutate used for treatment in this cohort of patients was synthesised by two different methods depending on the type of ^177^LuCl_3_ that was used. For carrier-added ^177^LuCl_3_ (IDB, Holland), synthesis was performed using the manual bench-top method. For non-carrier-added ^177^LuCl3 (ANSTO, Sydney, Australia, or ITG, Munich, Germany), synthesis was performed using the Eckert & Ziegler Modular Lab PharmTracer automated synthesis system.

Patients that had completed all cycles of treatment with full imaging performed at each of the 4 cycles were considered for review. Full imaging consisted of quantitative SPECT/CT with the kidneys in the centre of the axial field of view (FoV) at time points 4, 24 and 96–120 h after injection. Image quantification included CT-derived corrections for scatter and attenuation [14] as well as application of a camera-specific dead time correction (performed on each projection) and sensitivity factor [15]. All data were acquired on an Intevo SPECT/CT system (Siemens Healthineers, Hoffman Estates, USA) with crystal thickness 15.8 mm, using medium energy collimators and a 20% width energy window centred on the 208 keV photopeak. The X-ray CT data was acquired with automatic exposure control at a 130 kV tube voltage with no contrast. A calibration standard was also placed in the SPECT/CT FoV to allow for validation of quantification accuracy.

Dose estimates were derived by defining kidney volumes of interest (VOIs) on the co-registered CT data and using the calculated percent injected activity at each time point to fit time-activity curves (TACs) in OLINDA-EXM [16]. The appropriate adult model (male or female) was chosen with correction for patient-specific kidney mass derived from the segmented CT. The data were scaled by the net-injected activity for each cycle to arrive at total absorbed renal dose (in units of Gy) using full imaging data. Various methods were then explored as alternatives when three imaging time points at all cycles are not available for analysis, and results from each compared to full imaging data values.

### Method 1

Half of the available patient cohort was used to derive an average renal clearance half-time (*t*_1/2_ average). This was then applied to the other half of the cohort assuming they had only 4 h imaging acquired. As such, the 24 h and 120 h imaging data were estimated by applying *t*_1/2_ average to the 4 h percent injected activity value, and all time points used in OLINDA-EXM to estimate dose to kidneys at each cycle.

### Method 2

As above, however, this method assumed the second cohort had only 24 h imaging acquired, and pseudo-4 h and − 120 h time points were derived through application of *t*_1/2_ average. All time points were then used in OLINDA-EXM to estimate dose to kidneys at each cycle.

### Method 3

The complete imaging data for cycle 1 were used to derive a patient-specific renal clearance half-time (*t*_1/2_ patient) for each patient. This was then applied to subsequent cycles for that patient assuming they only had 4 h imaging acquired. As such, the 24 h and 120 h imaging data were estimated by applying *t*_1/2_ patient to the 4 h percent injected activity value, and all time points used in OLINDA-EXM to estimate dose to kidneys at cycles 2, 3 and 4.

### Method 4

As for method 3, however, this method assumed that a patient’s cycle 2, 3 and 4 had only 24 h imaging acquired. Pseudo-4 h and − 120 h time points were derived through the application of *t*_1/2_ patient, and all time points were then used in OLINDA-EXM to estimate dose to kidneys at cycles 2, 3 and 4.

### Method 5

The complete imaging data for cycle 1 were used to derive the percentage of injected activity (%IA) present at 120 h as a fraction of that at 24 h. Subsequent cycles were assumed to have only 4 and 24 h imaging available, which were scaled by the derived fraction to create a pseudo-120 h time point. All time points were then used in OLINDA-EXM to estimate dose to kidneys at cycles 2, 3 and 4.

Comparison of each method with the gold standard approach using all available image data was assessed with Bland-Altman analysis based on relative differences. The relative difference in derived absorbed dose estimates between each method and the gold standard approach were calculated, and the standard deviation was estimated treating variability at both the intra- and inter-patient level equally. Whilst the absorbed doses across the total 80 cycles of therapy are not strictly independent, given the relatively high number of degrees of freedom, this was thought to be a valid assumption for statistical interpretation. The limits of agreement were defined as the average difference ± twice the standard deviation (2SD).

## Results

A total of 20 complete courses of treatment from 18 different patients (8 females, 10 males), resulting in a total of 80 cycles of therapy, were included in the analysis. The average and standard deviation of the injected radioactivity per cycle of treatment was 7756 ± 418 MBq. The average renal absorbed dose per cycle of treatment was measured as 2.9 ± 1.4 Gy, or 0.38 ± 0.19 Gy/GBq of injected Lutate. The average renal clearance half-time derived from the first 40 cycles of therapy included in the study was 54.2 ± 12.6 h, or 53.0 ± 15.6 h from the complete cohort. This was derived through a mono-exponential fit to the data.

**Fig. 1:**
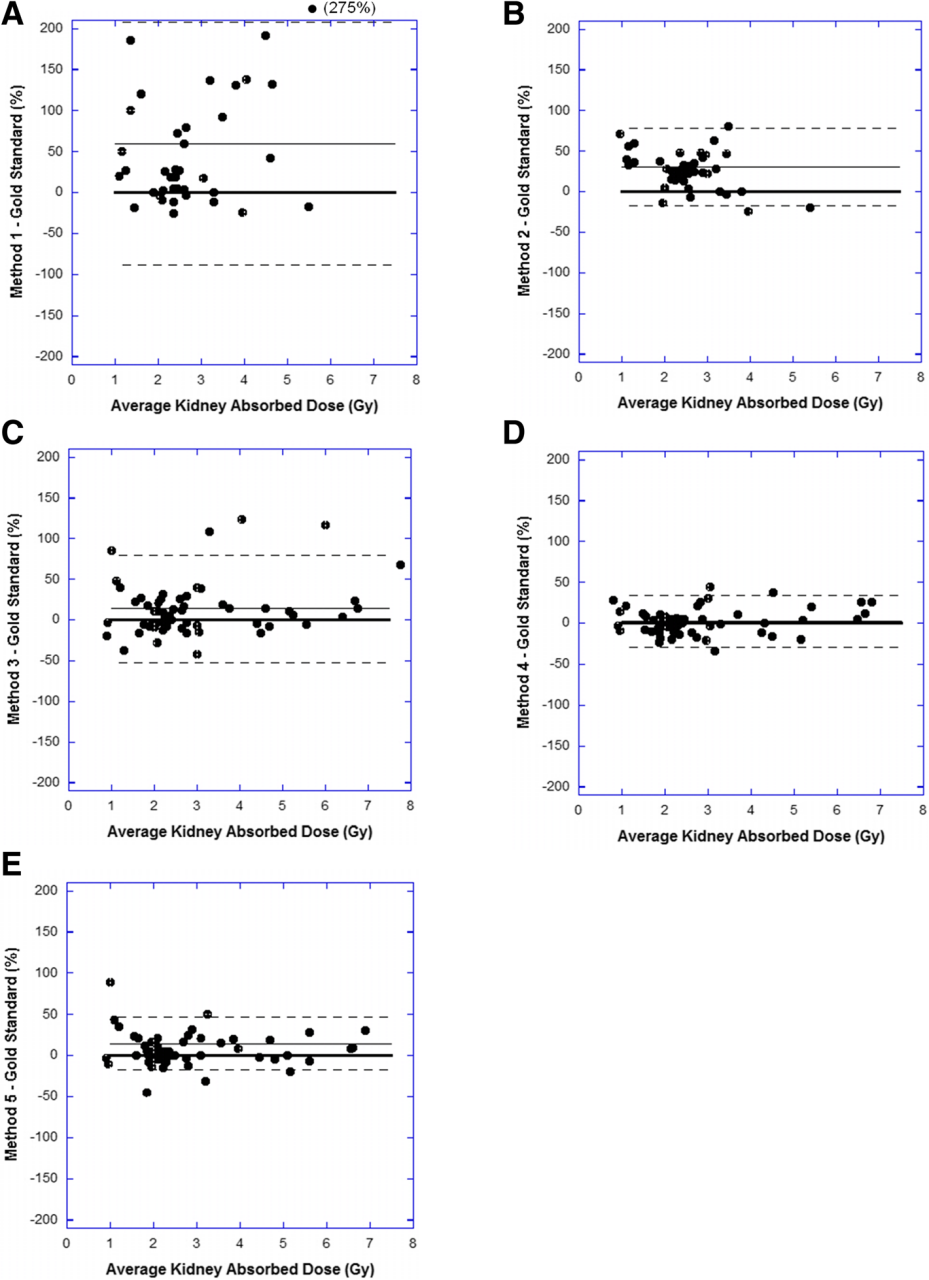
Modified Bland-Altman plots comparing the calculated renal absorbed dose (Gy) of each method to the gold standard approach, when complete imaging data (three time points) is available. Dashed lines correspond to the limits of agreement (2SD). Solid lines correspond to the average difference between the methods; heavy solid lines correspond to zero difference

Table 1 represents the results from each method when comparing the absorbed renal dose derived from complete data (treated as the gold standard) with the method-specific estimated values. Figure 1 represents the difference between each method and the gold standard approach through a modified Bland-Altman analysis, with relative differences on the y-axis and average kidney absorbed dose from the two methods on the x-axis. The use of a population average half-time in method 1 and method 2 appear to demonstrate a systematic overestimate of kidney absorbed dose when compared to the gold standard approach and resulted in the largest differences with limits of agreement of 59 ± 138% and 30 ± 38%, respectively. In this case, imaging at 24 h post-injection and using *t*_1/2_ average to estimate both 4 h and 120 h time point data (method 2) leads to more accurate dose estimates when compared to using *t*_1/2_ average to scale 4 h imaging only (method 1). Similarly, the use of 4 h imaging only in conjunction with a cycle 1 derived patient-specific half-time (method 3) demonstrated relatively large random differences with limits of agreement 14 ± 68%. The method that achieved results most consistent with the gold standard approach was method 4 (the use of full imaging at cycle 1 and 24 h imaging at cycles thereafter) resulting in limits of agreement of 2 ± 30%. The use of full data on cycle 1 to estimate 120 h time point data on subsequent cycles (method 5), i.e. two imaging time points still required, also produced acceptable estimates of kidney dose with limits of agreement of 14 ± 30%.

**Table 1:** Difference values (as a percentage) between various methods with sub-complete data when compared to the full data set analysis (gold standard method); method 1: *t*_1/2_ average applied to 4 h data; method 2: *t*_1/2_ average applied to 24 h data; method 3: *t*_1/2_ patient from cycle 1 applied to 4 h data on subsequent cycles; method 4: *t*_1/2_ patient from cycle 1 applied to 24 h data on subsequent cycles; method 5: cycle 1 %IA at 120 h applied to subsequent cycles with only 4 h and 24 h data acquired

Method	*N*	Average difference (%)	Standard deviation (%)	Maximum difference (%)	Median difference (%)
1	40	59	69	275	26
2	40	30	19	80	27
3	60	13	34	132	8
4	60	2	16	45	1
5	60	14	16	89	10

*N* the number of cycles analysed to derive difference values

## Discussion

In Australia, most patients receiving Lutate therapy do so on an out-patient basis. Given the large distance for referrals to specialist NET centers, this can make temporal imaging for dosimetry purposes challenging. Even in the case of centers who keep patients in following therapy for radiation protection purposes, prolonged camera time may not be easily accessible or optimal for patients comfort. If clinical and/or patient logistics do not allow more than one imaging time point to be performed on any cycle, then the use of an average patient clearance time is best applied for scaling of a 24 h acquisition as opposed to a 4 h acquisition. Application to a sole late time point (120 h) acquisition was not investigated as, in our experience, this is the most difficult time point for patients to return for imaging. The measured average renal dose in this study is in keeping with other SPECT/CT-based estimates presented in the literature, being slightly higher than that of Claringbold [17] (0.3 Gy/GBq) and lower than average estimates from Sandstrom [18, 19] (0.7 and 0.6 Gy/GBq, respectively), with variations likely due to quantification and dosimetry methods, in addition to study cohorts and site-specific administration technique. The average renal clearance half-time of 53.0 h in our cohort is in good agreement with that presented by Garske et al [13] (52.6 h averaged across both kidneys at both early and late cycles), Sundlöv et al. (51.6 h) [20], and Bergsma et al. (58 h) [11, 21]. However, given the large average deviations of methods 1 and 2 from the gold standard estimates made when using complete data sets (59% and 30%, respectively), it may be that the use of an average population renal half-time in conjunction with a single time point of image data will not yield accurate estimates of kidney absorbed dose. This finding is in contrast to Sundlöv’s results [8] which demonstrated reasonably consistent estimates of renal clearance between patients suggesting a population average clearance time to be a robust method. However, the authors have recognised that this could in part be due to the uniformity of patient selection required under the trial setting.

If complete data (3 imaging time points) can be acquired at cycle 1, it appears that a single imaging time point at 24 h at subsequent cycles is adequate to estimate renal dose. This method (method 4) can be expected to lead to, on average, a deviation of 2% from dose values that would have been acquired under full data set conditions and offers a good compromise between patient-specific dosimetry and management of clinical camera time as well as demands on the patient. This result is in keeping with the findings of Garske et al [13], who concluded that in most patients it was safe to estimate absorbed dose from 24 h activity concentration measures assuming a constant effective half-life during therapy. The use of a single 24 h image at later cycles is more accurate than the use of a single imaging time point at 4 h (average deviation of 13%); however, it is recognised that scanning patients while they are already in the department following their Lutate infusion may be more appealing for some sites, particularly for patients who do not live locally, and so this method may still be deemed acceptable if 24 h imaging is not available. If two imaging time points (4 and 24 h) are acquired on subsequent cycles as opposed to just a single imaging time point (4 or 24 h), and the %IA at 120 h estimated based on the first cycle data, there seems to be no gain in accuracy of dose estimates (method 5). In the case of this data, the use of a patient-specific clearance time derived through an exponential fit of cycle 1 imaging data resulted in more accurate dose estimates when compared to estimating the 120 h %IA alone. These findings may in part be due to the fitting method used, with the 4 h time point most likely still in the first phase of uptake and in some cases deviating from the fitted TAC. In addition, it is recognised that some centers use a late imaging time point that is beyond 120 h post-injection, and the use of such data may result in the various methods explored in this study having either larger or smaller deviations from reference values.

Given the assumptions involved in dosimetry analysis, ranging from limitations of scanner spatial resolution to the model assumptions of patient size and geometry, the deviations demonstrated when utilising full imaging at cycle 1 to inform kinetics at subsequent cycles would seem acceptable (methods 3, 4 and 5, in this study). In addition to easing the burden on department facilities and patients, the approach of lesser imaging time points also has benefits in the reduction of patient radiation exposure associated with multiple CT scans. It should certainly be noted that patient renal function, generally measured at stages before, after and during cycles of treatment, will also have a bearing on clearance rate and kidney dose [22]. As such, if a significant change in renal function is noted during the treatment phase after the first cycle, patients should ideally have full imaging data re-acquired and modelled at later cycles.

## Conclusions

This study suggests that dose estimates made from single time point imaging are feasible and more accurate when derived from 24 h as opposed to 4 h single time point imaging. Ideally, a full data set should be acquired at cycle 1 to derive a patient-specific renal clearance half-time, as opposed to the use of an average patient renal clearance half-time.

## Abbreviations

^177^Lu: Lutetium-177
CT: (X-ray) computed tomography
FoV: Field of view
IA: Injected activity
SPECT: Single photon emission computed tomography
*t*_1/2_ average: Average clearance half-time derived from first half of patient data sets
*t*_1/2_ patient: Patient-specific clearance half-time derived from first cycle of treatment imaging
TAC: Time-activity curve
VOI: Volume of interest
